# Supplementary material for: Drosophila RASopathy models identify disease subtype differences and biomarkers of drug efficacy
Source: iScience. 2021 Mar 13;24(4):102306. doi: 10.1016/j.isci.2021.102306 (PMC8026909; doi:10.1016/j.isci.2021.102306)
Supplement: Document S1. Transparent methods and Figures S1–S10 [file mmc1.pdf]

iScience, Volume *24*

Supplemental information

Drosophila RASopathy models identify disease subtype differences and biomarkers of drug efficacy

Tirtha K. Das, Jared Gatto, Rupa Mirmira, Ethan Hourizadeh, Dalia Kaufman, Bruce D. Gelb, and Ross Cagan

Supplemental Data

<u>Supplemental Figures</u>

Supplemental Figure 1

A

	Type of Mutation	Syndrome/ Disease
dRAF1-S259A	Activating	NS
hRAF1-S257L	Activating	NS
hRAF1-D486G	Inactivating	NS
hRAF1-L613V	Activating/GOF	NS
hBRAF-W531C	Activating	NS
hKRAS-G12D	Activating/GOF	NS/CFCS/cancers
hHRAS-G12S	Activating/GOF	CS/cancers
hPTPN11-D61G	Activating/GOF	NS/JMML
hPTPN11-Y279C	Inactivating	NSML
hPTPN11-N308D	Activating/GOF	NS
hPTPN11-R498W	Dominant Neg.	NSML
hPTPN11-Q510P	Dominant Neg.	NSML
hPTPN11-Q510E	Dominant Neg.	NSML

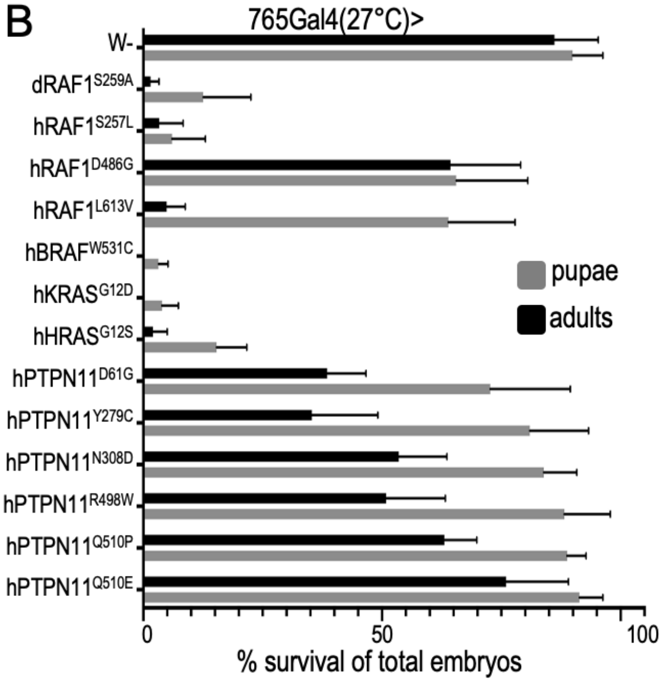

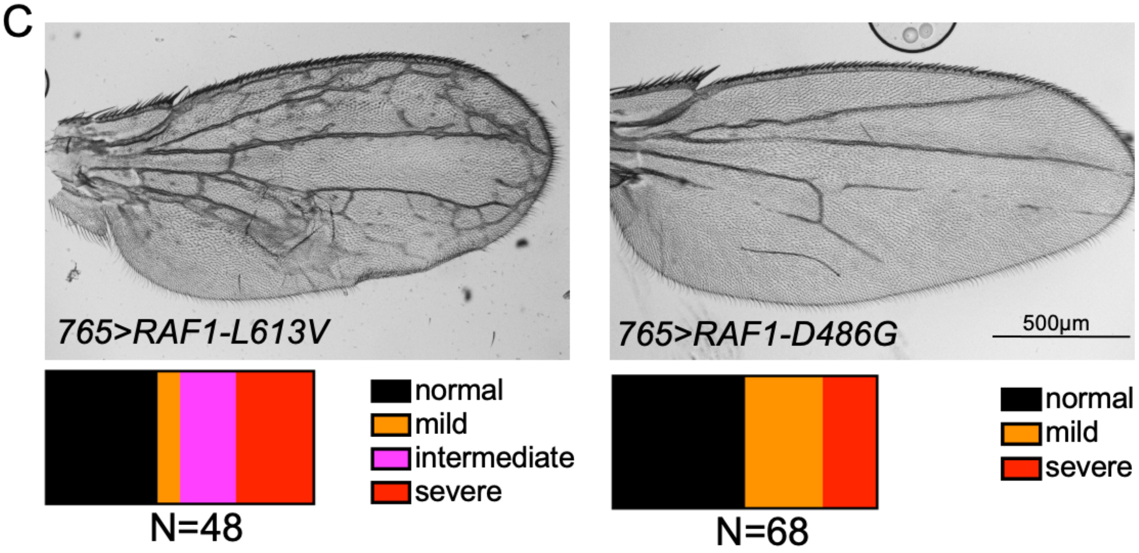

Supplemental Figure 1. Modeling RASopathy isoforms, Related to Figures 1, 2

(A) List of RASopathy variants and fly models developed in this study, the nature of the mutations, and the syndromes/diseases they are associated with in patients (Tartaglia and Gelb, 2005, 2010; Chan, Kalaitzidis and Neel, 2008; Hijikata *et al.*, 2017; Zheng *et al.*, 2018). JMML- Juvenile myelomonocytic leukemia; NS – Noonan syndrome; NSML-Noonan syndrome with multiple lentigens; CFCS – cardiocutaneous syndrome; CS – Costello syndrome.

(B) Viability assay results for each RASopathy model at 27 °C. For each condition four replicates were analyzed and error bars represent standard error of the mean (SEM) here and in subsequent figures; see Methods. The percentage of surviving pupa are shown as grey bars and adults as black bars.

(C) Bright field images of adult fly wings in which RASopathy isoforms *RAF1*^{L613V} and *RAF1*^{D486G} were expressed uniformly throughout the wing epithelia using *765-GAL4*. After adults eclosed, their wings were analyzed and the severity of wing defects assessed and binned into four categories as indicated. Finally, the proportion of each phenotype was assessed by analyzing the indicated number of wings and is represented as a color-coded bar. Similar analysis was performed with all RASopathy isoforms in Supplemental Figures 2-5. *RAF1*^{L613V} expression led to ectopic wing veins across the entire wing, while *RAF1*^{D486G} expression suppressed wing vein formation consistent with nature of the mutation inactivating the catalytic activity of RAF1.

Supplemental Figure 2

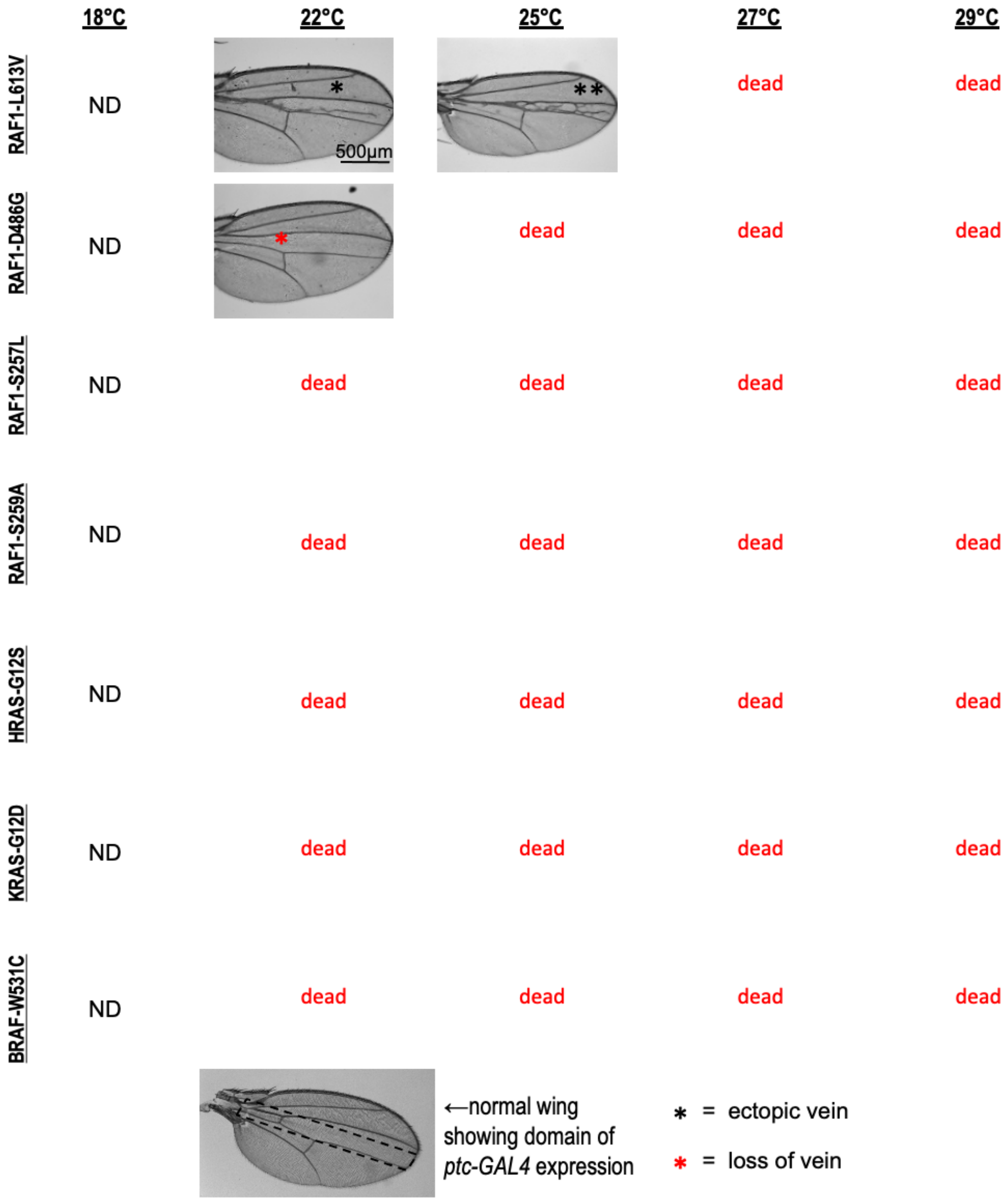

Supplemental Figure 2. *765>RAS/RAF* isoforms altered wing venation, animal viability, related to Figure 2

Bright field images of adult fly wings in which RAS/RAF RASopathy isoforms were overexpressed using the *ptc-GAL4* driver. The control wing at the bottom includes a dotted outline indicating the region within which *ptc-GAL4* is active. GAL4 activity progressively increases at higher temperature, leading to increased transgene expression and stronger lethality and venation phenotypes. *ptc>RASopathy* embryos were collected and grown at indicated temperatures; if adults eclosed, then their wings were analyzed. Black asterisk indicates ectopic veins and red asterisk indicates loss/suppression of normal veins.

Supplemental Figure 3

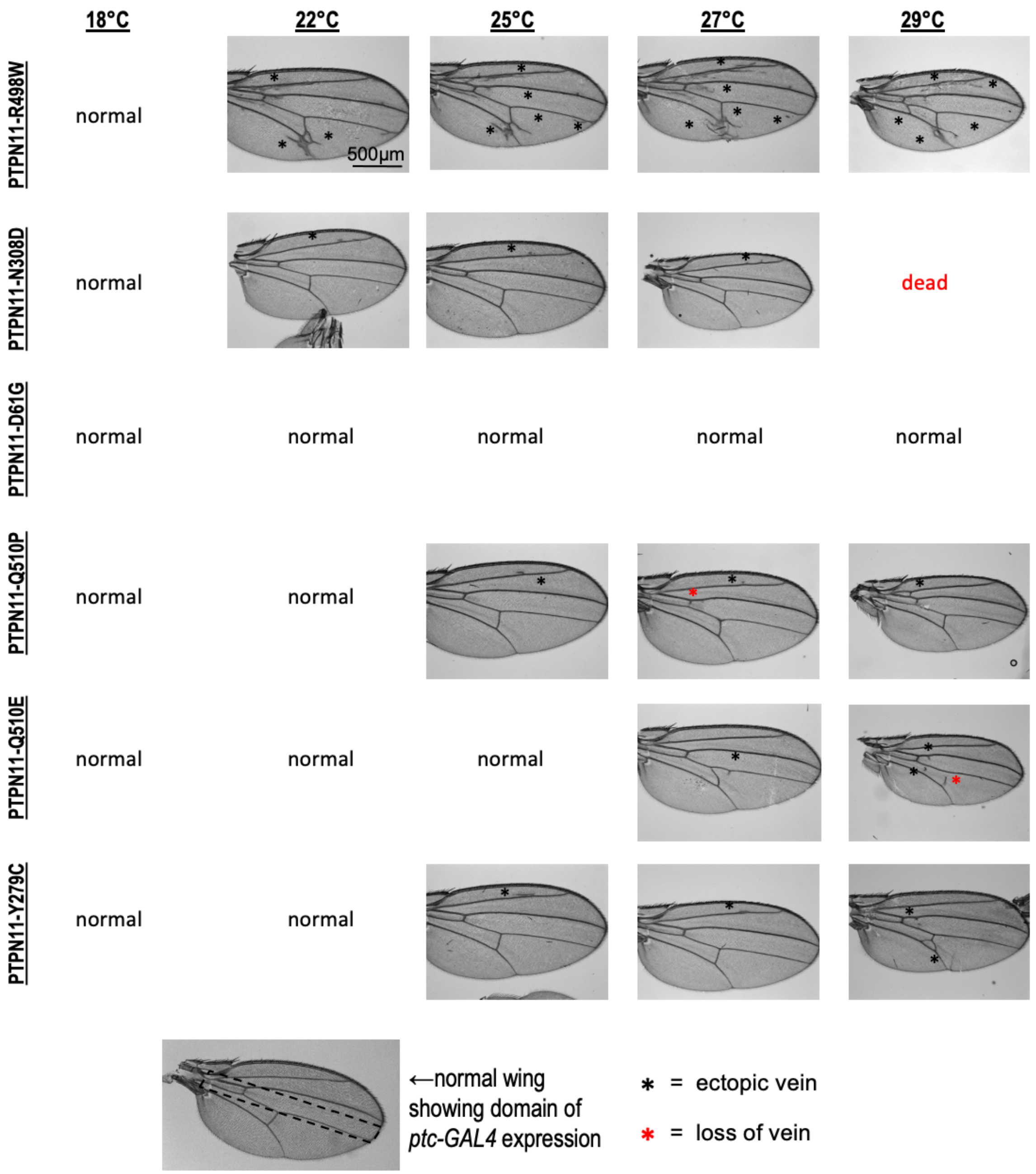

Supplemental Figure 3. *ptc>PTPN11* isoforms altered wing venation, animal viability, related to Figure 2

Bright field images of adult fly wings in which human *PTPN11* RASopathy isoforms were overexpressed using the *ptc-GAL4* driver. The control wing at the bottom includes a dotted outline indicating the region within which *ptc-GAL4* is active. GAL4 activity progressively increases at higher temperature, leading to increased transgene expression and stronger lethality and venation phenotypes. *ptc>RASopathy* embryos were collected and grown at indicated temperatures and, if adults eclosed, then their wings were analyzed. Black asterisk indicates ectopic veins and red asterisk indicates loss/suppression of normal veins.

Supplemental Figure 4

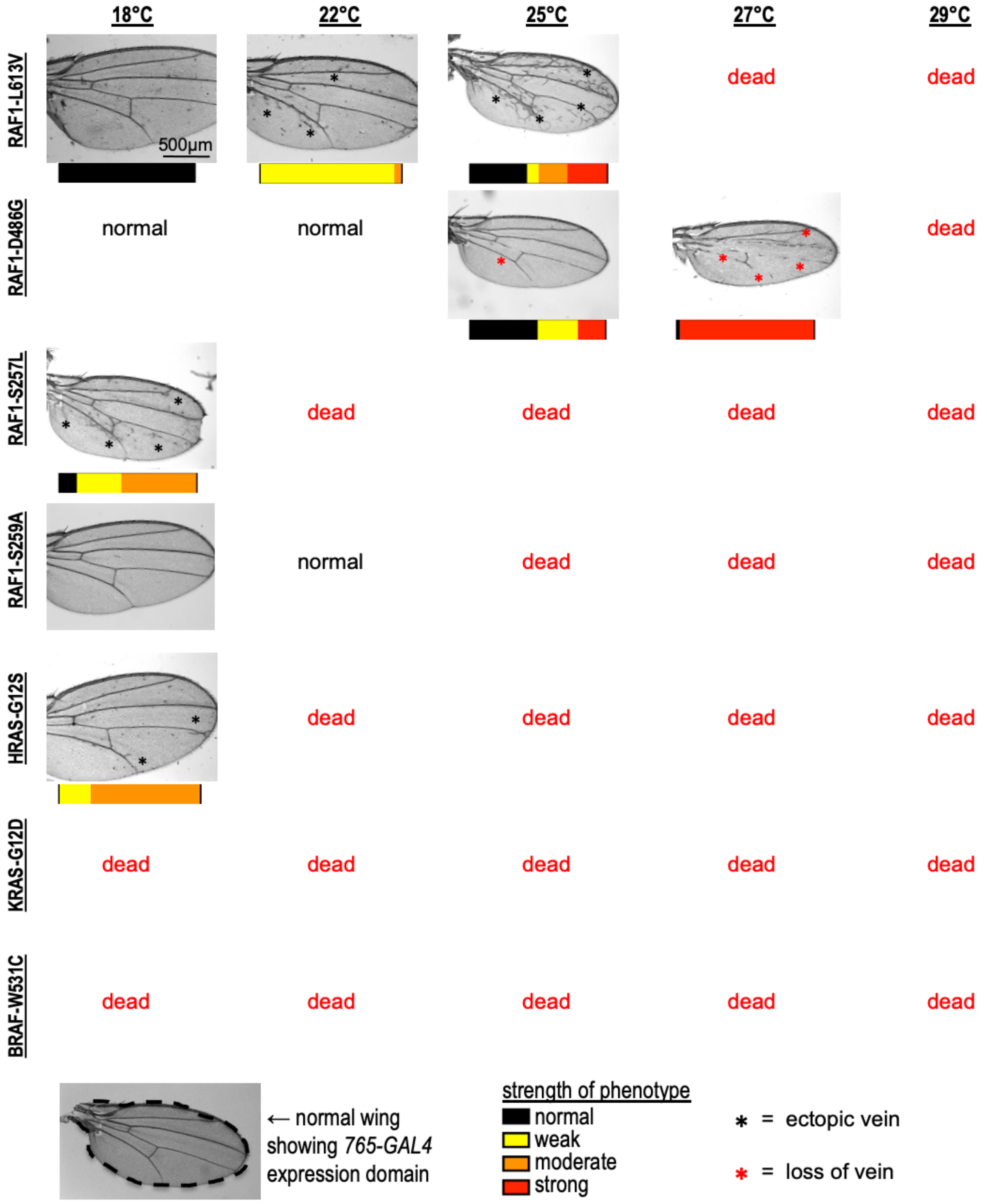

Supplemental Figure 4. *765>RAS/RAF* isoforms altered wing venation, animal viability, related to Figure 2

Bright field images of adult fly wings in which RAS/RAF RASopathy isoforms were overexpressed using a *765-GAL4* driver. The control wing at the bottom includes a dotted outline indicating the region within which *765-GAL4* is active. GAL4 activity progressively increases at higher temperature, leading to increased transgene expression and stronger lethality and venation phenotypes. *765>RASopathy* embryos were collected and grown at indicated temperatures and, if adults eclosed, then their wings were analyzed. Penetrance and proportion of phenotypes indicated by color coded bar below some experiments. Black asterisk indicates ectopic veins and red asterisk indicates loss/suppression of normal veins.

Supplemental Figure 5

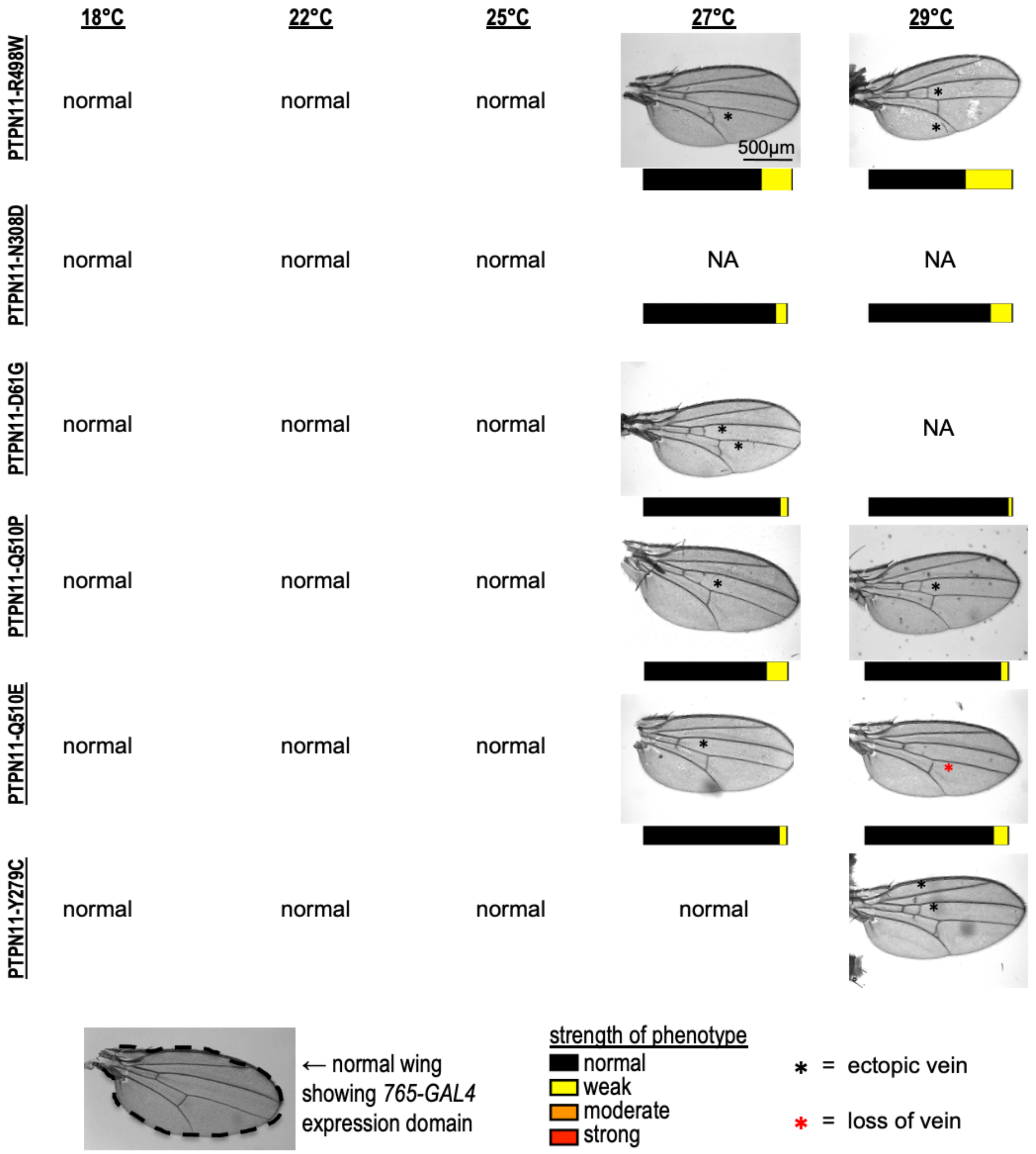

Supplemental Figure 5. *765>PTPN11* isoforms altered wing venation, animal viability, related to Figure 2

Bright field images of adult fly wings in which *PTPN11* RASopathy isoforms were overexpressed using the *765-GAL4* driver. The control wing at the bottom includes a dotted outline indicating the region within which *ptc-GAL4* is active. GAL4 activity progressively increases at higher temperature, leading to increased transgene expression and stronger lethality and venation phenotypes. *765>Rasopathy* embryos were collected and grown at indicated temperatures and, if adults eclosed, wings were analyzed. Penetrance and proportion of phenotypes indicated by color coded bar below some experiments. Black asterisk indicates ectopic veins; red asterisk indicates loss/suppression of normal veins.

Supplemental Figure 6

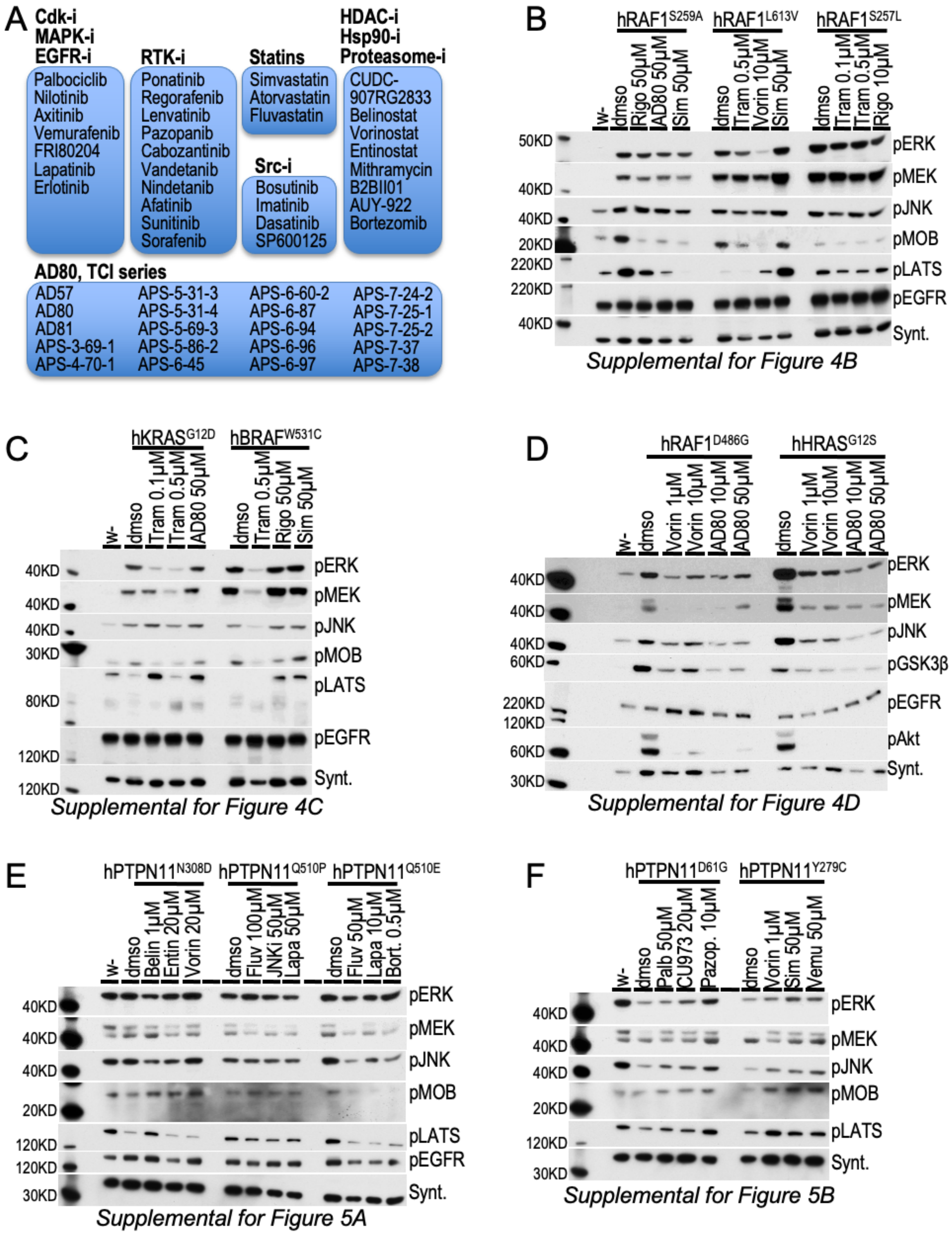

Supplemental Figure 6. List of drugs, compounds used in this study, and size markers for related Westerns. Related to Figures 3, 4, 5

A. List of drugs and compounds used to screen the different RASopathy models for increased pupa and adult viability using *765-GAL4* at 27 °C as described in Figure 4. A subset of drugs that showed high efficacy in the viability screen were used for analysis of pathway activation and signaling analysis using *tubulin-GAL4*;*gal80^{ts}* at 27 °C as described in Figures 4, 5. B-F. Westerns providing 1-2 size markers that match main figure panels as indicated.

Supplemental Figure 7

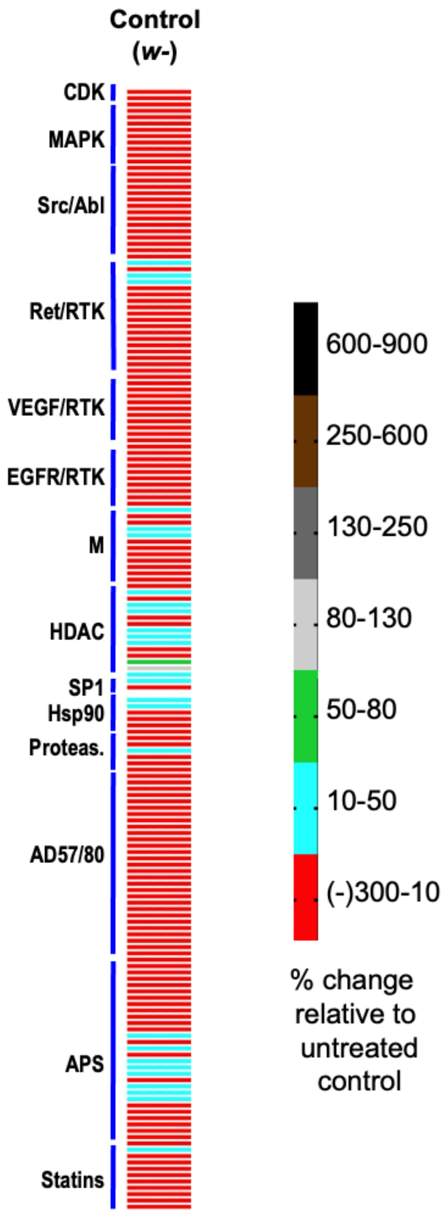

Supplemental Figure 7. Summary of drug response in control animals, related to Figure 3

Heat map depicting response of control (wild type genotype) flies to a panel of indicated drugs and tool compounds used to screen against the RASopathy models analyzed in this study in Figure 3. The heat map indicates the ratio of the number of pupae surviving following treatment compared to no treatment controls. This is represented as percentage change compared to control as shown in adjacent key. As in Figure 3, viability is assessed as the mean of four replicates for each condition. Each model exhibited a unique pattern of responses to the panel of drugs tested. The AD57/AD80 and APS family of tool compounds were developed in-house as previously published.

Supplemental Figure 8

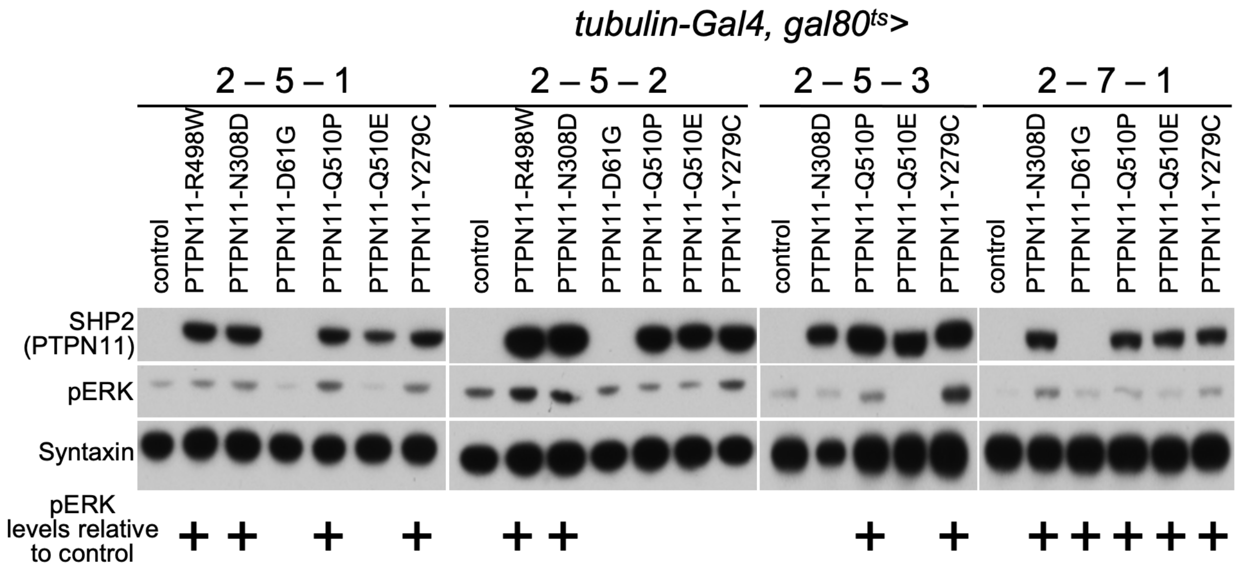

Supplemental Figure 8. Western blot analysis of PTPN11 transgenic models, related to Figure 5

Western blot analysis of different growth, collection, and induction conditions (see Figure 4A) for analysis of pathway activation by *PTPN11* isoforms. Whole larval lysates were collected from different *PTPN11* lines under the indicated conditions. Western blot analysis performed to detect MAPK pathway activation. Some isoforms upregulated pERK levels in the 2-5-1 condition but downregulated it in the 2-5-2 condition (-Q510P and –Y279C). This suggests feedback-induced downregulation of upstream MAPK components such as phosphorylated ERK (pERK), a common feature of the MAPK pathway. Other isoforms also show complex regulation of pERK but all isoforms increase pERK compared to control in the 2-7-1 condition. The SHP2 (*PTPN11*) antibody detected mostly equivalent levels of expression across *PTPN11* lines. –D61G was not detected using the SHP2 antibody, perhaps due to mutation-induced loss of the antibody epitope; its presence was confirmed by genomic PCR of fly models.

Supplemental Figure 9

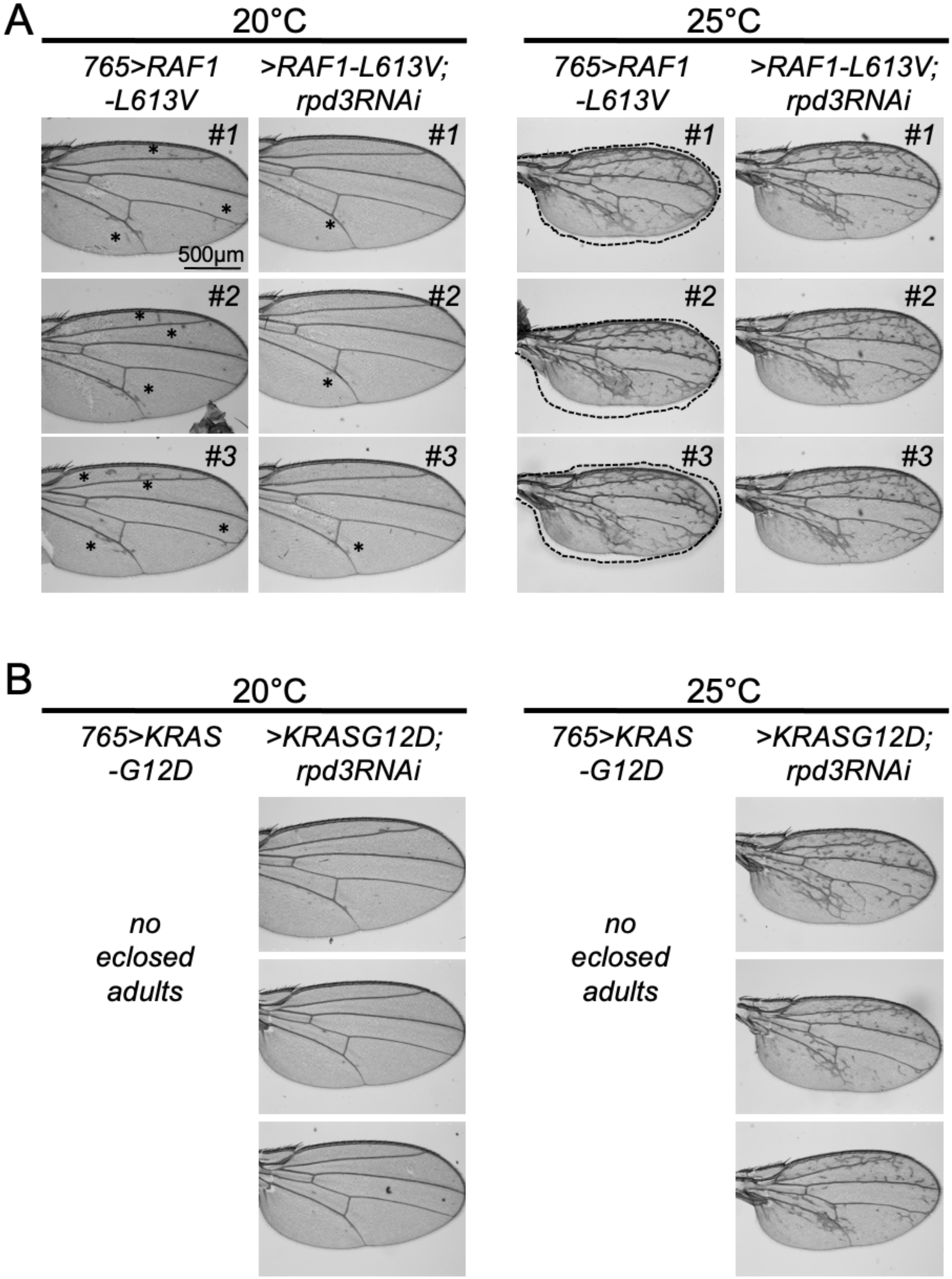

Supplemental Figure 9. Functional analysis of the impact of reducing rpd3 on *765>RAS/RAF* wing venation, related to Figure 6

(A) Genetic modifier experiments with *RAF1*^{L613V} isoforms show dependency on HDAC1. Each isoform was expressed throughout the developing larval wing disc using the *765-GAL4* driver under different temperature conditions. *765>RAF1*^{L613V} flies exhibited ectopic wing venation phenotypes 20 °C, which was suppressed by RNAi-mediated knockdown of the fly HDAC1 ortholog Rpd3 (*765>RAF1*^{L613V}, *rpd3-RNAi*). The suppression of ectopic wing venation did not occur at 25 °C with stronger induction of the isoform; interestingly at 25 °C, knockdown of Rpd3 consistently altered wing size as shown by dotted outline of the *765>RAF1*^{L613V},*rpd3-RNAi* wing superimposed on *765>RAF1*^{L613V} wing images. Black asterisk indicates ectopic veins. Three wings of each experiment are shown for comparison.

(B) Genetic modifier experiments with the *KRAS*^{G12D} isoform demonstrated functional dependency on HDAC1 for ectopic wing venation. When *765>KRAS*^{G12D} flies were raised at 20 °C and 25 °C no adults eclose; this developmental lethality was suppressed by knockdown of *Rpd3*, resulting in adult eclosure. At 20 °C, *765>KRAS*^{G12D}*,rpd3-RNAi* flies exhibited near-normal wing vein patterning. At 25 °C the ectopic wing venation pattern was not suppressed, again presumably due to stronger induction of the *KRAS*^{G12D} transgene. Three wings for each experiment are shown for comparison.

Supplemental Figure 10

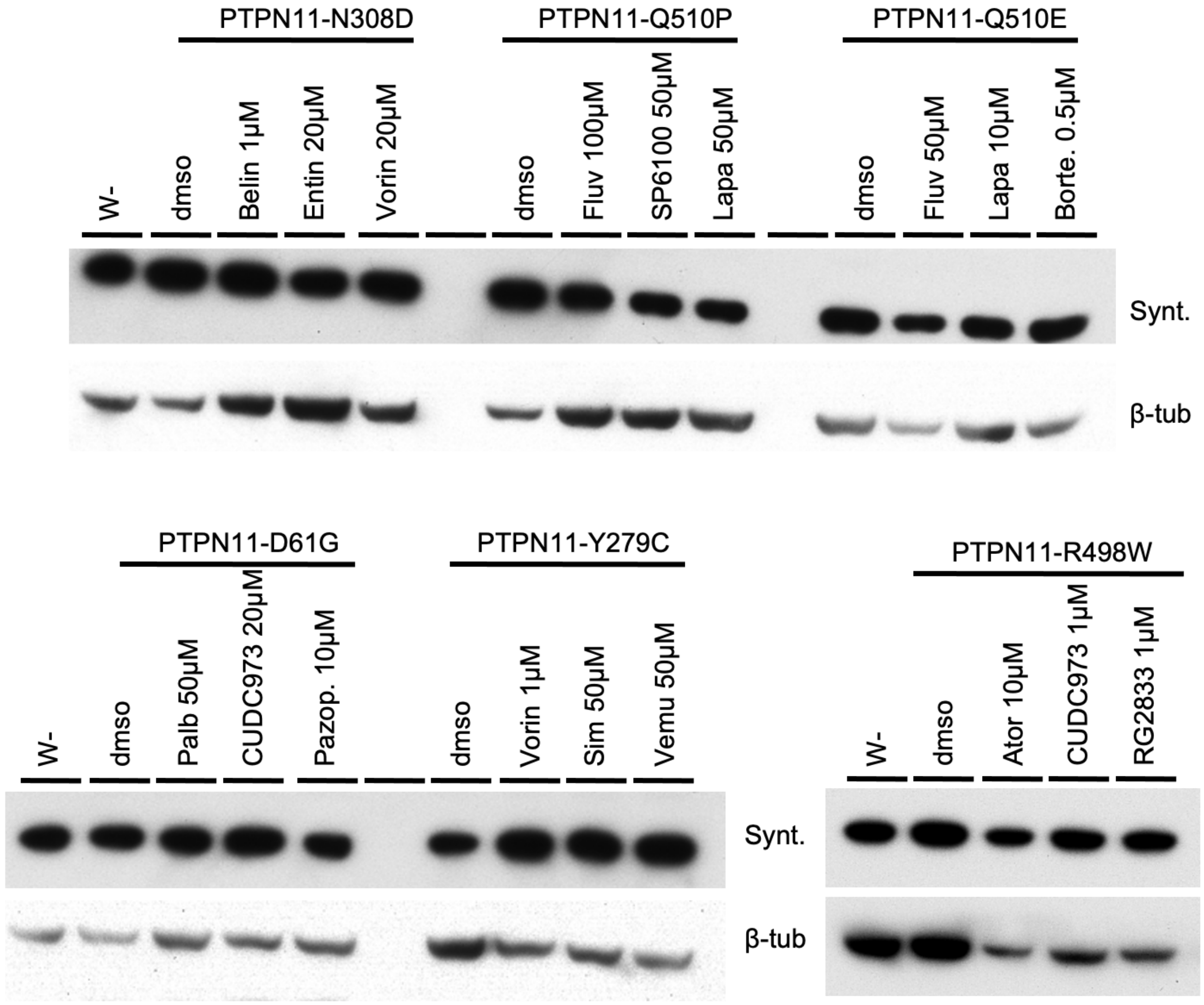

Supplemental Figure 10. Western blot analysis of PTPN11 lines, related to Figures 3, 4, 5, 6

Western blot analysis of indicated RASopathy models analyzed in this study. The first lane represents lysates from *w-* control flies; dmso represents treatment with the solvent in the absence of drug. Drug doses represent the condition at which the drugs showed efficacy in the drug screens in Figure 3, in western blot analyses in Figures 4 and 5, and summary in Figure 6C. While each lane was carefully normalized for equivalent total protein loading based on a BIORAD assay, traditional loading control markers like syntaxin and ß-tubulin often did not track with each other; this suggested complex effects of the RASopathy variants on these housekeeping genes.

Supplement- Transparent Methods

Antibodies

Antibodies used for Drosophila western blot analysis were: anti-pJNK; anti-pAKT; anti-pMOB; anti-pEGFR; anti-pMEK; anti-pLATS; anti-pGSK3β - (Cell Signaling); anti-pERK - (Sigma); anti-Actin; anti-Syntaxin; anti-ß-tubulin - (Developmental Studies Hybridoma Bank). Except anti-Syntaxin, all other antibodies were developed against human protein and various previous studies, by our group as well as others, have shown they cross react and can identify the Drosophila protein.

Comprehensive Statistical Analysis

For pupal and adult viability analysis in Figure 1, mean and standard error of the mean (SEM) were calculated and 4-5 vials/experiment (biological replicates) per condition were analyzed. Each vial contained between 20-80 developing embryos. For the large ~60 drug library screen in Figure 4, four vials per drug (biological replicates) with approximately 20–40 embryos per vial were analyzed by aliquoting a slurry of collected embryos in embryo compatible buffer (Das, Dana, *et al.*, 2013; Na *et al.*, 2013). For this large screen, absolute numbers of surviving pupae/adults were compared to no drug treatment to obtain a ratio of drug treatment/no drug treatment (percentage increase over baseline). To assess statistical significance of difference between means, t-Test with Welch's correction was performed using PRISM software. The correction was used to account for samples with unequal variances and unequal sample sizes. Candidate drugs showing the highest efficacy (drug conditions with p values ranging from 0.05 to 0.3) in this primary screen were re-tested in a secondary test in a similar manner, except in addition total number of starting embryos in each vial was visually counted. This allowed for precise quantitation of increased viability by comparing the proportion of pupa/embryos rescued, with or without drug. Statistical significance, $p<0.05$, of difference between means, t-test with Welch's correction was performed using PRISM software in these retests.

Generating Drosophila Transgenic RASopathy Models

The cDNAs corresponding to RAS/MAPK pathway gene variants found in patients were subcloned into Drosophila transformation vector pUAST-attB. cDNA's were obtained from commercially available (AddGene) sources and variants were introduced by performing overlapping PCR. Internal primers with altered DNA sequence were combined with primers at the start and end of the corresponding cDNA which contained restriction enzyme overhangs. Injection and creation of attp40 transgenics was done by BESTGENE Inc. Flies corresponding to *UAS-Rpd3* knockdown lines (TRIP lines) were obtained from Bloomington Drosophila Stock Center. The following primers were used:

hRAF1(S257L)
F-hRAF1-kzk-EcoR1-Start
GAATTCCAAAACATGGAGCACATACAGGGAGCTTGG
F-hRAF1-767(S257L)
GGTTGACATCCACACCTAATGTCCAC
R-hRAF1-756(S257L)
CATTAGGTGTGGATGTCAACCTCTGCCTCTGG
R-hRAF1-XbaI-Stop
TCTAGACTAGAAGACAGGCAGCCTCGGGG

hRAF1(L613V)
F-hRAF1-kzk-EcoR1-Start
GAATTCCAAAACATGGAGCACATACAGGGAGCTTGG
F-hRAF1-1835(L613V)
CTGTACCGAAGATCAACCGGAGCGC
R-hRAF1-1822(L613V)
GTTGATCTTCGGTACAGAGTGTTGGAGCAG
R-hRAF1-XbaI-Stop
TCTAGACTAGAAGACAGGCAGCCTCGGGG

hRAF1(D486G)
F-hRAF1-kzk-EcoR1-Start
GAATTCCAAAACATGGAGCACATACAGGGAGCTTGG
F-hRAF1-1454(D486G)
GAGGTTTTGGTTTGGCAACAGTAAAGTC
R-hRAF1-1437(D486G)
TGCCAAACCAAAACCTCCAATTTTCACTGTTAAG
R-hRAF1-XbaI-Stop
TCTAGACTAGAAGACAGGCAGCCTCGGGG

hBRAF(W531C)
F-hBRAF-kzk-NotI-Start
GCGGCCGCCAAAACATGGCGGCGCTGAG
F-hBRAF-1651(W531C)
GTGTTGTGAGGGCTCCAGCTTGTATC
R-hBRAF-1637(W531C)
GGAGCCCTCACAACACTGGGTAACAATAGC
R-hBRAF-XbaI-Stop
TCTAGATCAGTGGACAGGAAACGCACCATATCC

hKRAS(G12D)
F-hKRAS-kzk-NotI-Start
GCGGCCGCCAAAACATGACTGAATATAAACTTGTGGTAGTTGGAGCTGATGGCG
R-hKRAS-XhoI-Stop
GAGCTCTTACATAATTACACACTTTGTCTTTGAC

hHRAS(G12S)
F-hHRAS-kzk-NotI-Start
GCGGCCGCCAAAACATGACGGAATATAAGCTGGTGGTGGTGGGCGCCTCCGGTGT
R-hHRAS-XbaI-Stop
TCTAGATCAGGAGAGCACACACTTGCAGCTCATGCAGCCGGG

hPTPN11(D61G)
F-hPTPN11-kzk-EcoR1-Start
GAATTCCAAAACATGACATCGCGGAGATGGTTTC
F-hPTPN11-179(D61G)
GTGGTTACTATGACCTGTATGGAGGG
F-hPTPN11-164(D61G)
CATACAGGTCATAGTAACCACCAGTGTTCTGAATC

R-hTPTN11-XbaI-Stop
TCTAGATCACAGATCCTCTTCAGAGATGAGTTTTCTG

hPTPN11(Y279C)
F-hPTPN11-kzk-EcoR1-Start
GAATTCCAAAACATGACATCGCGGAGATGGTTTC
F-hPTPN11-833(Y279C)
GATGTAAAAACATCCTGCCCTTTGAT
R-hTPTN11-825(Y279C)
CAAAGGGCAGGATGTTTTTACATCTATTTTTG
R-hTPTN11-XbaI-Stop
TCTAGATCACAGATCCTCTTCAGAGATGAGTTTTCTG

hPTPN11(N308D)
F-hPTPN11-kzk-EcoR1-Start
GAATTCCAAAACATGACATCGCGGAGATGGTTTC
F-hPTPN11-919(N308D)
GCAGATATCATCATGCCTGAATTTGAAAC
R-hPTPN11-907(N308D)

R-hTPTN11-XbaI-Stop
TCTAGATCACAGATCCTCTTCAGAGATGAGTTTTCTG

hPTPN11(R498W)
F-hPTPN11-kzk-EcoR1-Start
GAATTCCAAAACATGACATCGCGGAGATGGTTTC
F-hPTPN11-1498(R498W)
GTGTGGTCTCAGAGGTCAGGGATG
R-hTPTN11-1479(R498W)
ACCTCTGAGACCACACCATCTGGATG
R-hTPTN11-XbaI-Stop
TCTAGATCACAGATCCTCTTCAGAGATGAGTTTTCTG

hPTPN11(Q510E)
F-hPTPN11-kzk-EcoR1-Start
GAATTCCAAAACATGACATCGCGGAGATGGTTTC
F-hPTPN11-1525(Q510E)
GCAGAGTACCGATTTATCTATATGGCG
R-hTPTN11-1513(Q510E)
GATAAATCGGTACTCTGCTTCTGTCTGGAC
R-hTPTN11-XbaI-Stop
TCTAGATCACAGATCCTCTTCAGAGATGAGTTTTCTG

hPTPN11(Q510P)
F-hPTPN11-kzk-EcoR1-Start
GAATTCCAAAACATGACATCGCGGAGATGGTTTC
F-hPTPN11-1525(Q510P)
GCACCGTACCGATTTATCTATATGGC
R-hTPTN11-1513(Q510P)

GATAAATCGGTACGGTGCTTCTGTCTGGAC
R-hTPTN11-XbaI-Stop
TCTAGATCACAGATCCTCTTCAGAGATGAGTTTTCTG

Inhibitor Studies in Flies

Drugs were obtained from LC laboratories or Selleck Chemicals and were dissolved in dmso as stock solutions ranging from 1-200mM. Drugs (500–1000 µl) were diluted in molten (~50–60 °C) enriched fly food, aliquoted into 5-ml vials to obtain the final drug concentration in food. Based on previous analysis, after consumption of drug-containing food by larvae the circulating concentration of drug is 100–1000 fold lower than the concentration in fly food (Bangi *et al.*, 2016; Das, Esernio and Cagan, 2018). 30–60 embryos of each genotype were raised on drug-containing food until they matured as third-instar larvae (whole larvae for western blot assay) or allowed to proceed to adulthood (viability assay and wing vein quantitation assay).

Western Blot of Whole Larval Lysates and Quantitation

Three third-instar larva of each genotype (*tubulin-GAL4*; $gal80^{ts}$ > *UAS-transgene*) were dissolved in Lysis Buffer (50 mM Tris, 150 mM NaCl, 1% Triton-X100, 1 mM EDTA) supplemented with protease inhibitor cocktail (Sigma) and phosphatase inhibitor cocktail (Sigma). Total protein in each sample was quantitated using BIORAD protein assay. Total protein amounts in each lysate was established by performing Bradford assay (BIORAD), and equivalent amounts (2–10 µg) of total protein was loaded per lane. As many of the isoform activated pathways are known to act on housekeeping proteins such as Syntaxin, Actin, and Tubulin, we relied on initial protein quantitation for accurate loading (Das, Dana, *et al.*, 2013; Das, Sangodkar, *et al.*, 2013; Das and Cagan, 2017; Das, Esernio and Cagan, 2018). During western blot development we assessed, when possible, all three markers mentioned above. As expected, we found that different loading controls (Syntaxin *vs*. tubulin) were regulated differentially by RASopathy variant activation, and therefore initial protein quantitation (Bradford/BIORAD) is the more reliable method of ensuring accurate loading (Supplemental Figure 10). Samples were resolved on Invitrogen NU-PAGE gradient SDS-page and transferred by standard protocols. Membranes were stripped with SIGMA Restore stripping buffer and reprobed with other antibodies to assess signal under exactly the same loading conditions. Exposed films were scanned and the western signal for each marker (TIFF files) was quantitated using the densitometric analysis in Image J.

Whole Mount Imaging of Fly Wings

For adult wing vein analysis, wings were dissected and kept in 100% ethanol overnight, mounted on slides in 80% glycerol in phosphate buffered saline solution, and imaged by regular light microscopy using Leica DM5500 Q microscope.
